# Degradation Mechanism of Perfluoroelastomer (FFKM) in the Acidic SC2 Solution of Semiconductor Radio Corporation of America (RCA) Cleaning

**DOI:** 10.3390/polym17223081

**Published:** 2025-11-20

**Authors:** Fandi Meng, Xiaolong Wen, Qishan Chen, Tao Zhang, Li Liu

**Affiliations:** Corrosion and Protection Center, Northeastern University, Shenyang 110819, China

**Keywords:** perfluoroelastomer (FFKM), SC2, degradation mechanism, TAIC

## Abstract

The degradation behavior and mechanism of perfluoroelastomer (FFKM) in the acidic Standard Clean 2 (SC2) solutions were studied to facilitate their application in semiconductor Radio Corporation of America (RCA) cleaning processes. The results indicate significant degradation of the mechanical properties of FFKM in the SC2 solution, characterized by surface pitting and particle formation, accompanied by progressive destruction of the cross-linked network. FTIR and XPS analyses revealed that degradation primarily occurs in the Trialkyl isocyanurate (TAIC) cross structure, while the main chain and side groups remain stable. HCl-induced acid hydrolysis and H_2_O_2_-induced oxidation act synergistically to break down the cross-link structures. This degradation compromised the filler matrix interface, leading to filler release and a consequent progressive deterioration of the overall properties of FFKM. This work elucidates the degradation mechanism of the FFKM in acidic environments, providing a scientific basis for the reliable design and lifetime prediction of FFKM components in semiconductor wet processes.

## 1. Introduction

Perfluoroelastomer (FFKM) is extensively employed as a critical sealing component in semiconductor manufacturing owing to its exceptional thermal stability and chemical resistance to aggressive media [1,2,3,4]. This outstanding stability primarily stems from the high chemical inertness conferred by the dense encapsulation of both the FFKM backbone and side chains with high-energy C–F bonds (485 kJ/mol) [5]. However, as device dimensions shrink and integration levels increase, process requirements for ultra-clean environments and material reliability have become increasingly stringent [6,7]. Even minor degradation of sealing materials can trigger leakage, particle contamination, and chemical leaching, thereby impacting yield rates and equipment stability [8,9,10,11]. Consequently, a thorough understanding of degradation mechanisms of FFKM under typical semiconductor operating conditions is particularly essential [3,12].

Previous studies have investigated FFKM degradation under elevated temperatures and various plasma environments. Estefanía et al. [13] examined FFKM degradation at 200 °C and 300 °C and reported that primary chain scission was accompanied by secondary cross-linking and macroscopic softening/swelling after prolonged aging. For medium to high temperature conditions, Zhuo et al. [14] investigated the degradation of FFKM with a Trialkyl isocyanurate (TAIC) cross-linking structure, and demonstrated that the C–N bonds within the cross-linking structure represent thermal weak points prone to fracture, leading to disruption of the cross-linked network. Concerning plasma environments, Nicholas et al. [9] and Tetsuya Goto et al. [15] tested FFKM using CF_4_/O_2_ and O_2_/Ar plasmas, respectively. They established the degradation behavior of FFKM under plasma conditions and confirmed that plasma radicals are the primary drivers of surface changes in the elastomer. Despite the high chemical inertness and tolerance of FFKM across diverse environments, significant degradation persists following prolonged service under severe conditions, with distinct degradation mechanisms evident across different environments [16,17]. Therefore, further investigation into the service behavior of FFKM in specific semiconductor fabrication environments is essential for mitigating material failure risks.

During semiconductor fabrication, steps involving solvents (including amines), corrosive acids, and alkalis are commonplace for etching, rinsing, cleaning, or stripping unwanted materials and contaminants from wafer surfaces. In addition to high temperature and plasma processes, the chemical media employed in these steps can erode elastomeric seals, causing swelling and degradation, or leach metallic impurities that compromise chamber cleanliness [18,19]. The Radio Corporation of America (RCA) cleaning process is a wet chemical cleaning technique developed in 1965 by Kern, Puotinen, and others at the RCA Laboratories in Princeton, New Jersey, USA. It is primarily employed for the removal of contaminants from the surfaces of semiconductor silicon wafers [20]. It is chiefly divided into two stages: Standard Cleaning 1 (SC1) utilizes an alkaline peroxide system (NH_4_OH/H_2_O_2_/H_2_O) to remove organic matter and particulates; and Standard Cleaning 2 (SC2) employs an acidic peroxide system (HCl/H_2_O_2_/H_2_O) to remove metal ions and form a mild passivation layer on the wafer surface [20,21]. Both materials contain highly corrosive substances, which significantly affect the elastic sealant. With respect to the alkaline SC1 solution, research has elucidated the differential effects of NH_4_OH and H_2_O_2_ on the cross-linked structure of FFKM, confirming the competitive behavior between these agents during FFKM degradation [22]. For acidic solutions, Han et al. [23] investigated the mechanical properties and creep behavior of fluoroelastomers in hydrochloric acid environments. The results indicate that immersion in hydrochloric acid causes significant swelling of the fluoroelastomer, rendering it softer and more brittle. This outcome strongly contrasts with the behavior of FFKM in NH_4_OH solutions. In other words, although both the SC1 and SC2 solutions contain the strongly oxidizing H_2_O_2_ component, the differing effects of NH_4_OH and HCl may account for the markedly distinct performance of the FFKM in these solutions. Consequently, further investigation into FFKM degradation behavior within SC2 solutions is warranted.

Typically, the cleaning temperature for the SC2 process is maintained between 75 and 85 °C, with each treatment step lasting approximately 10–15 min [20,24]. Therefore, to better approximate actual operating conditions and investigate the cumulative effects on materials after multiple cleaning cycles, this study subjected FFKM with a TAIC crosslinking structure to a 28-day service test in an 85 °C SC2 solution. Concurrently, to distinguish potential differential effects attributable to HCl versus H_2_O_2_ in the solution, comparative analyses were conducted using separate 85 °C HCl and H_2_O_2_ solutions. Macroscopic performance changes in samples before and after immersion were evaluated through tensile testing, mass change measurements, and surface morphology analysis. Chemical structural alterations were characterized using Fourier Transform Infrared Spectroscopy (Nicolet iS10, Thermo Fisher Scientific, Waltham, MA, USA), X-ray Photoelectron Spectroscopy (XSAM 800, Kratos Analytical Ltd., Manchester, UK), and Dynamic Mechanical Analysis (Q800, TA Instruments, New Castle, DE, USA). These findings indicate that HCl hydrolyses the N–C and N–C(=O)–N bonds within the TAIC structure, facilitating solution penetration and exposing reactive sites. H_2_O_2_ subsequently initiates oxidative chain scission, accelerating the disruption and relaxation of the cross-linked network. This synergistic interaction weakens the filler-matrix interface bonding, prompting the desorption of carbon black and the formation of voids, which ultimately leads to a significant deterioration of the material’s mechanical properties.

## 2. Materials and Methods

### 2.1. Sample Preparation

**Figure 1:**
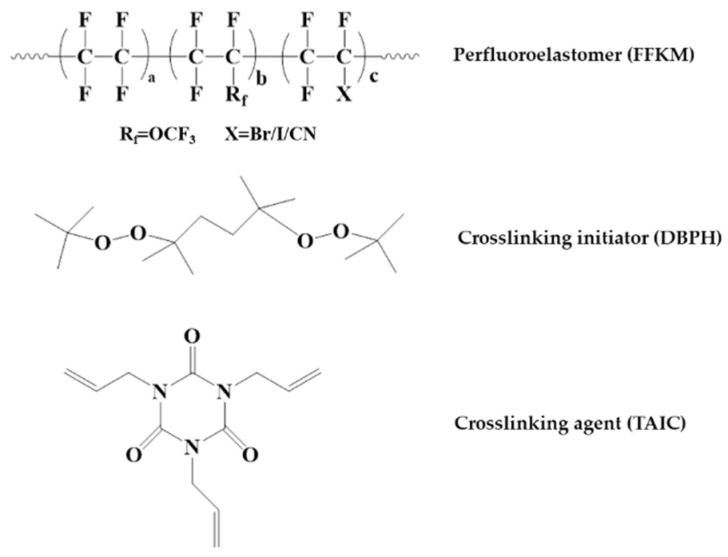
The chemical structures of the materials employed in this study: Perfluoroelastomer (FFKM), crosslinking initiator (DBPH), and crosslinking agent (TAIC).

The FFKM samples used in this study were prepared in the laboratory. The specific process involved emulsion polymerization of perfluoromethylethylene oxide (PMVE), tetrafluoroethylene (TFE), and a sulfur-containing third monomer (CSM) in a high-pressure reactor at 60 °C and 4 MPa. Chain transfer agents were employed to control the molecular weight and rheological properties. Following the completion of the reaction, the polymer was frozen and condensed to obtain pellets. These pellets were washed with deionized water, vacuum-dried at 100 °C, and adequately plasticized to produce raw rubber. The raw rubber was vulcanized with 2,5-Dimethyl-2,5-di(tert-butylperoxy)hexane (DBPH) and TAIC, using carbon black as filler, and the relevant chemical structure is shown in Figure 1. The weight ratio of components was 100 parts green rubber, 15 parts filler, and 2 parts cross-linking agent. TFE, PMVE, DBPH, and TAIC were supplied by ZiGong HongChuan Chemical Additives Factory (Zigong, China). N990 carbon black was purchased from McLin Company (Elk Grove, CA, USA). Ethanol, hydrochloric acid solution (37 wt.%), and hydrogen peroxide (30 wt.%) were obtained from China National Pharmaceutical Group Corporation (Beijing, China). The homemade sample shapes included two types: dumbbell-shaped tensile samples conforming to the ASTM D412 standard [25] and rectangular samples measuring 10 mm × 10 mm × 2 mm.

### 2.2. Degradation Test

Before experimentation, the samples were cleaned with deionized water and anhydrous ethanol and then dried. The dried samples were placed within a reactor vessel containing the experimental solution, which was subsequently placed in an 85 °C oven for aging. Upon the conclusion of the experiment, the reactor vessel was removed and allowed to cool naturally to ambient temperature before sample extraction. The sample surfaces were cleaned with deionized water. The experimental solution was SC2 solution, with HCl (37 wt.%): H_2_O_2_ (30 wt.%): deionized water = 1:1:6. Based on the HCl: H_2_O_2_ ratio in SC2, a HCl solution control group was established (HCl solution composition: HCl (37 wt.%): deionized water = 1:6), and a H_2_O_2_ solution control group (H_2_O_2_ solution composition: H_2_O_2_ (30 wt.%): deionized water = 1:6) was established. The samples were immersed in each solution. The samples were immersed in each solution for designated periods of 7 days (short-term) and 28 days (long-term), with three replicates at each time point.

### 2.3. Physical and Chemical Characterization

#### 2.3.1. Mass

The test samples were measured using a precision balance (SQP, Sartorius, Göttingen, Germany; 0.01 mg readability) at room temperature. The mass of each sample was measured before and after immersion. Before each weighing, any excess solvent from the sample surface was wiped off with filter paper. The mass change in the sample was calculated via the following formula:

(1) $$M_{t}=\frac{m_{0,w}-m_{0}}{m_{0}}\times 100\%,$$

where *M_t_* denotes the mass change (%), *m*_0_ represents the mass of the sample before immersion, and *m*_0,*w*_ denotes the mass of the sample after aging for a period of time *t*.

#### 2.3.2. Micro-Structure

The surface morphology of the samples before and after aging was examined via a scanning electron microscope (JSM-IT800SHL, JEOL Ltd., Tokyo, Japan). Before examination, gold powder was sprayed onto the sample surfaces to increase the conductivity.

#### 2.3.3. ATR-FTIR

The surface chemical structural changes in the FFKM samples were analyzed with a Fourier transform infrared spectrometer (Nicolet iS10, Thermo Fisher Scientific, Waltham, MA, USA) in SMART ATR mode. The detection range was 650–4000 cm^−1^, with 4 cm^−1^ spectral resolution and 32 accumulated scans.

#### 2.3.4. XPS

The surface elemental changes in the FFKM samples were analyzed with X-ray photoelectron spectroscopy (XSAM 800, Kratos Analytical Ltd., Manchester, UK). By employing Al (mono) as the X-ray source, the acquired full XPS spectra were calibrated to C1s at 284.8 eV. 

#### 2.3.5. TGA

The thermal stability of the FFKM samples, both unaged and aged, was evaluated with a thermogravimetric analyzer (TG-DTA 8122, Rigaku Corporation, Tokyo, Japan). Samples (approximately 10 mg) in 70 μL aluminum oxide crucibles were heated from 30 °C to 800 °C at 20 °C/min under a nitrogen atmosphere to obtain TGA and DTG curves.

#### 2.3.6. TGA-FTIR

The TGA-FTIR system, consisting of a thermogravimetric analyzer (STA 8000, PerkinElmer Inc., Shelton, CT, USA) coupled to a Fourier transform infrared spectrometer (Frontier, PerkinElmer Inc., Shelton, CT, USA), was employed to analyze samples after aging in SC2 solution. FFKM samples were heated from 30 °C to 800 °C at 20 °C min^−1^ under a nitrogen atmosphere. The evolved gases from decomposition were transferred through heated lines into the FTIR gas cell for analysis.

#### 2.3.7. DSC

Differential scanning calorimetry (DSC) of the FFKM samples before and after aging was conducted via a differential scanning calorimeter (Discovery DSC2500, TA Instruments, New Castle, DE, USA). Under a nitrogen atmosphere, the samples were first heated to 80 °C and held isothermally for 5 min to eliminate the thermal history. They were then cooled at a rate of −10 °C/min to −60 °C and held isothermally for 5 min. Finally, the sample was heated from −60 °C to 100 °C at a rate of 10 °C/min, and the DSC curve was recorded during the final heating process.

#### 2.3.8. DMA

The storage modulus of the FFKM samples before and after aging was measured via a dynamic thermal mechanical analyzer (Q800, TA Instruments, New Castle, DE, USA). At 50 °C, an isothermal strain scan from 0.1% to 10% was performed at a constant frequency of 1.0 Hz under a static load of 0.01 N, employing a uniaxial tensile test mode. The measured storage modulus was calculated via the rubber elasticity theory formula (Equation (2)) [26,27] to determine the sample cross-link density. For filled rubber, corrections were applied according to the Guth–Smallwood model (Equation (3)) [28]:

(2) $$V_{e}=\frac{E_{0}}{3RT},$$

(3) $$E_{0}=\frac{E_{f}}{1+2.5\varphi_{f}+14.14\varphi_{f}^{2}},$$

where *V_e_* denotes the cross-link density of the rubber, *R* represents the gas molar constant (8.314 J·mol^−1^·K^−1^), *E*_0_ is the dynamic storage modulus of unfilled rubber, *T* is the absolute temperature, *E_f_* is the energy storage modulus of the filled rubber, and *φ_f_* is the volume fraction of the filler.

#### 2.3.9. Tensile Test

Tensile behavior was evaluated using a universal testing machine (AG-Xplus, Shimadzu Corporation, Kyoto, Japan; 100 kN). The samples were dumbbell-shaped tensile samples (in accordance with the ASTM D412 standard) [25], with the strain rate set at 500 mm/min.

## 3. Results

### 3.1. Mass Test

**Figure 2:**
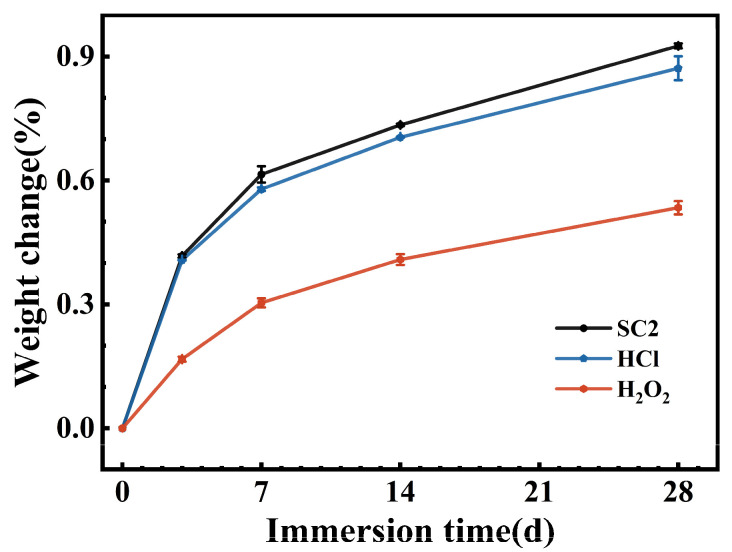
Plot of weight change results of FFKM after immersion in three solutions.

Figure 2 presents the variation in FFKM mass as a function of immersion time in three different solutions. All samples showed progressive weight increases, with a rapid uptake during the first 7 days that gradually leveled off thereafter. At 28 days, the absorption rates in the SC2, HCl, and H_2_O_2_ solutions were 0.92%, 0.87%, and 0.53%, respectively, indicating that SC2 had the highest absorption rate, followed by HCl, with H_2_O_2_ having the lowest absorption rate. Although FFKM may undergo mass-altering chemical reactions with some solutions, the figure demonstrates that such alterations are negligible. Notably, the greater weight change in the SC2 solution compared to HCl and H_2_O_2_ suggests that the weight change observed in the SC2 solution likely resulted from the combined effects of the HCl and H_2_O_2_ solutions.

### 3.2. Microscopic Morphology

**Figure 3:**
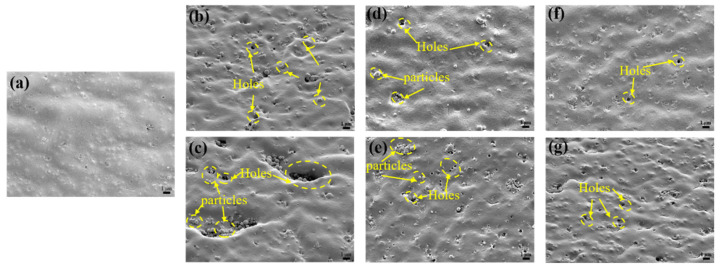
Surface morphology of FFKM (**a**) initially and after immersion in (**b**) SC2-7 d; (**c**) SC2-28 d; (**d**) HCl-7 d; (**e**) HCl-28 d; (**f**) H_2_O_2_-7 d; and (**g**) H_2_O_2_-28 d.

Figure 3 depicts the evolution of the surface morphology of FFKM after aging in various solutions. The original sample surface was flat and smooth without notable defects. After 7 days in SC2, small cavities and particulates appeared, which enlarged and aggregated markedly by day 28, revealing severe surface degradation. In the HCl solution, the samples exhibited a small number of cavities and particles at 7 days, with an increased quantity but a limited scale at 28 days. The samples aged in the H_2_O_2_ solution showed only minor surface roughening and a few small voids, with slight particle deposition over time. This is attributed to solution penetration disrupting the material cross-linking network, weakening the matrix’s retention of fillers [29,30]. Consequently, the fillers detached from the matrix and migrated to the surface. Among all environments, SC2 produced the most pronounced surface deterioration, followed by HCl, while H_2_O_2_ caused minimal alteration. This trend is consistent with the aforementioned mass change results, indicating that the synergistic action of HCl and H_2_O_2_ in SC2 solution accelerates the degradation of the FFKM surface morphology and filler precipitation.

### 3.3. Chemical Structural Evolution

**Figure 4:**
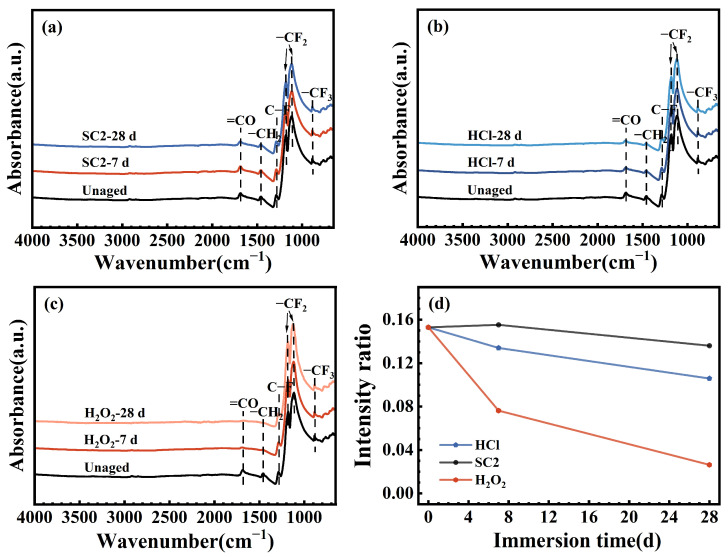
FTIR spectra of FFKM samples after immersion in (**a**–**c**) SC2, HCl, and H_2_O_2_ solutions and (**d**) the corresponding C=O/CF_2_ intensity ratio.

The infrared spectra of the FFKM samples after immersion in SC2, HCl, and H_2_O_2_ solutions for varying durations are presented in Figure 4a–c. The absorption bands at 1288 cm^−1^ (C–F stretching), 1184 cm^−1^ (symmetric CF_2_ stretching), 1115 cm^−1^ (asymmetric CF_2_ stretching), and 885 cm^−1^ (CF_3_ vibration) correspond to the FFKM polymer backbone [31]. Peaks located at 1684 cm^−1^ and 1463 cm^−1^ are attributed to the TAIC network, assigned to C=O and CH_2_ vibrations, respectively [32]. As shown in Figure 4a–c, after immersion in the three solutions, the CF, CF_2_, and CF_3_ signals showed negligible change, suggesting that the polymer backbone remained chemically stable. As shown in Figure 4d, the relative intensity of the characteristic C=O peak for the TAIC structure continuously decreased after immersion in HCl and H_2_O_2_ solutions. In contrast, no significant change was observed in the SC2 solution during the initial immersion period, with a gradual decrease occurring later. In the HCl solution, this change is attributed primarily to the hydrolysis of the TAIC cross-linked structure in a high-temperature acidic environment. Under these conditions, the oxygen atom on the TAIC ring undergoes protonation, leading to hydrolysis of the N–C(=O)–N bond into an N–C(–OH)–N bond. Consequently, the relative intensity of the C=O characteristic peak gradually decreases over time. In H_2_O_2_ solution, the TAIC ring structure undergoes oxidation by H_2_O_2_, converting N–C(=O)–N to HO–C(=O)–N. This transformation causes the relative intensity of the C=O characteristic peak to continuously decrease. In the SC2 solution, during the initial immersion phase, the N–C(=O)–N bonds in the TAIC cross-linked structure are converted into unstable HO–C(–OH)–N intermediates under the synergistic effects of HCl hydrolysis and H_2_O_2_ oxidation. These intermediates gradually decompose into C=O groups. Thus, the relative intensity of the C=O characteristic peak remained largely unchanged. However, during the later aging stage, the persistent destruction of C–N bonds in the TAIC ring structure by H_2_O_2_ led to hydrolysis of the crosslinked network, resulting in a gradual decrease in the relative intensity of the C=O characteristic peak.

**Figure 5:**
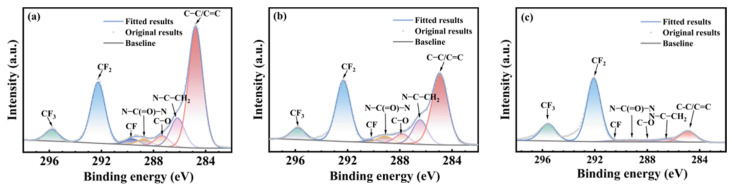
XPS C1s spectra of FFKM samples after aging in SC2 solution: (**a**) 0 d; (**b**) 7 d; (**c**) 28 d.

**Figure 6:**
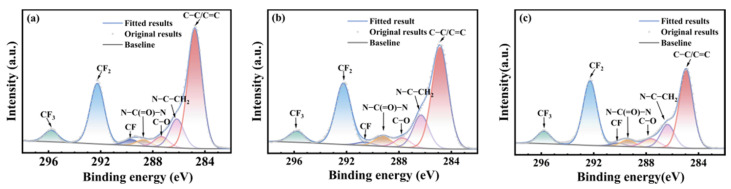
XPS C1s spectra of FFKM samples after aging in HCl solution: (**a**) 0 d; (**b**) 7 d; (**c**) 28 d.

**Figure 7:**
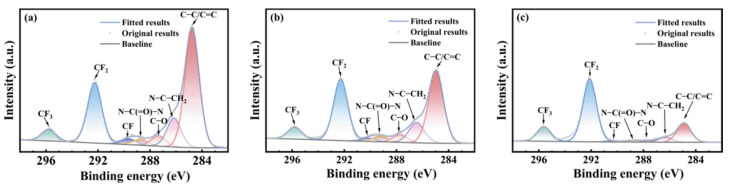
XPS C1s spectra of FFKM samples after aging in H_2_O_2_ solution: (**a**) 0 d; (**b**) 7 d; (**c**) 28 d.

Figure 5, Figure 6 and Figure 7 show the carbon atom (C1s) spectra of the FFKM samples after immersion in the SC2 solution, HCl solution, and H_2_O_2_ solution for different durations. The spectral components at 295.8 eV, 292.5 eV, 290.6 eV, 288.1 eV, and 284.9 eV correspond to carbon in CF_3_, CF_2_, CF, C–O–O–C, and C–C/C=C environments, respectively [31]. Additionally, the peak at 289.3 eV originates from the characteristic N–C(=O)–N carbon atom in the TAIC ring, whereas the peak at 286.8 eV belongs to the carbon atom bonded to nitrogen in the N–C structure outside the TAIC ring. Figure 5, Figure 6 and Figure 7 show that the C–C/C=C peak intensity in the FFKM samples gradually decreases with aging time in all three solutions, whereas the characteristic peaks associated with the main chain (CF_3_, CF_2_, CF, etc.) essentially remain unchanged. This confirms that the polymer backbone is chemically stable and that degradation predominantly affects the TAIC-derived crosslink network. In the HCl solution, the continuous decrease in the C–C/C=C peak intensity is attributed to the hydrolytic cleavage of N–C bonds outside the TAIC ring under high-temperature acidic conditions. In the H_2_O_2_ solution, the breakdown of C–N bonds within the TAIC ring structure and the progressive depletion of the residual cross-linking agent DBPH led to a relative decrease in the intensities of both the C–C–C–C=C characteristic peak and the TAIC characteristic peak. In the SC2 solution, HCl-induced hydrolysis and H_2_O_2_-induced C–N bond destruction of the TAIC ring structure occur simultaneously, further accelerating degradation of the material’s cross-linked structure. This results in a significant decrease in the relative intensity of the C–C/C=C bond characteristic peak after aging in the SC2 solution, indicating the most severe degradation.

**Figure 8:**
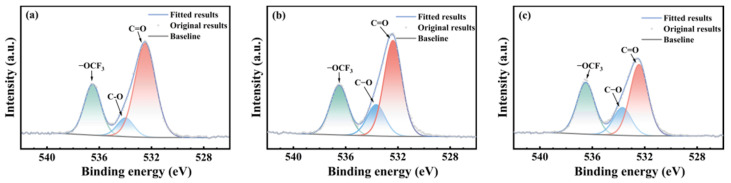
XPS O1s spectra of FFKM samples after aging in SC2 solution: (**a**) 0 d; (**b**) 7 d; (**c**) 28 d.

**Figure 9:**
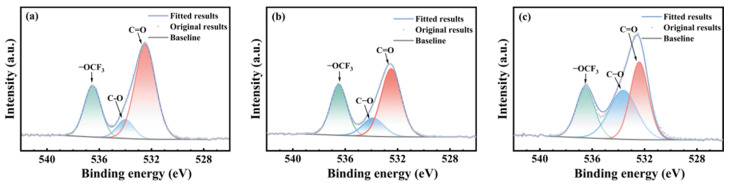
XPS O1s spectra of FFKM samples after aging in HCl solution: (**a**) 0 d; (**b**) 7 d; (**c**) 28 d.

**Figure 10:**
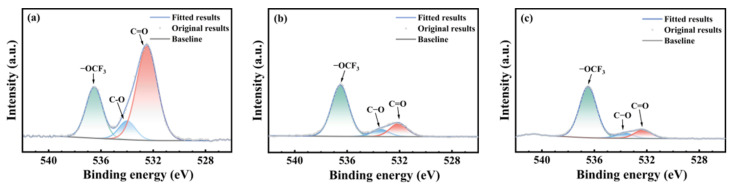
XPS O1s spectra of FFKM samples after aging in H_2_O_2_ solution: (**a**) 0 d; (**b**) 7 d; (**c**) 28 d.

Figure 8, Figure 9 and Figure 10 present the oxygen atom (O1s) spectra of FFKM samples after 28 days of immersion in SC2 solution, HCl solution, and H_2_O_2_ solution, respectively. For the unaged samples, the binding energies at 536.5 eV, 534.0 eV, and 532.4 eV correspond to oxygen atoms in the –OCF_3_, C–O, and C=O structures, respectively [33]. After immersion, the evolution of C–O and C=O components differs among the media but is consistent with ATR-FTIR trends. In the HCl solution, the relative intensity of the C–O peak gradually increased, whereas that of the C=O peak decreased, which was attributed primarily to hydrolysis of the TAIC cross-linked structure under high-temperature acidic conditions. In the H_2_O_2_ solution, the N–C(=O)–N group in the TAIC ring structure was oxidized by H_2_O_2_, converting it to HO–C(=O)–N, resulting in a continuous decrease in the relative intensity of the C=O characteristic peak. In the SC2 solution, during the initial immersion stage, the N–C(=O)–N bonds in the material cross-linked structure are converted into unstable HO–C(–OH)–N intermediates under the synergistic action of HCl and H_2_O_2_, which subsequently decompose into C=O. Consequently, the relative intensity of the C=O characteristic peak shows no significant change, whereas the relative intensity of the C–O bond characteristic peak gradually increases. During the later immersion stage, continuous H_2_O_2_ degradation of C–N bonds in the TAIC structure caused cross-linked degradation, leading to decreased relative intensities of both the C=O and C–O characteristic peaks. After immersion in all three solutions, the relative intensity of the –OCF_3_ characteristic peak corresponding to the material side chain showed almost no change, indicating that the FFKM side chain remained stable and was largely unaffected by the solutions.

### 3.4. Thermal Stability

**Figure 11:**
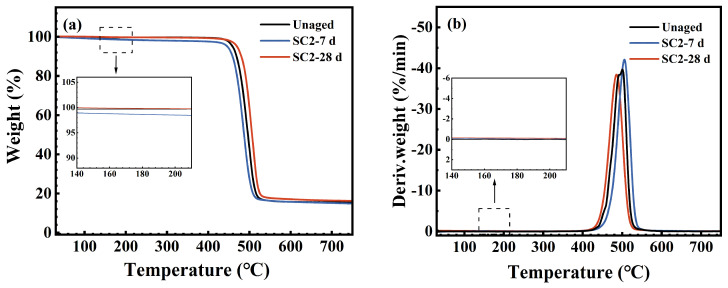
(**a**) TG; (**b**) DTG curve of FFKM after aging in SC2 solution.

**Figure 12:**
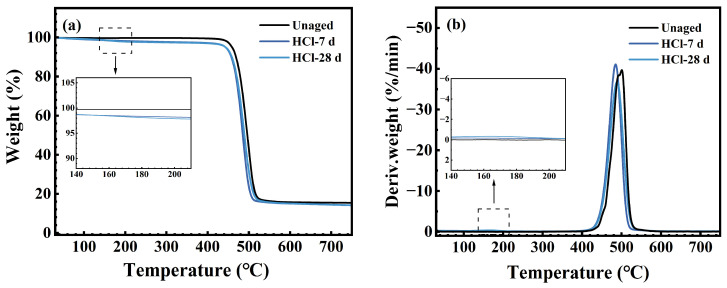
(**a**) TG; (**b**) DTG curve of FFKM after aging in HCl solution.

**Figure 13:**
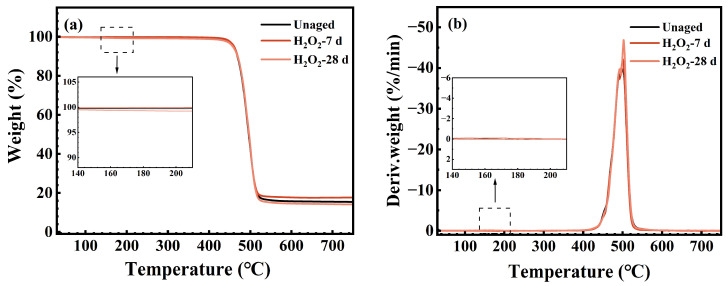
(**a**) TG; (**b**) DTG curve of FFKM after aging in H_2_O_2_ solution.

Figure 11, Figure 12 and Figure 13 display the TG and DTG curves of the FFKM samples after immersion in the SC2 solution, HCl solution, and H_2_O_2_ solution, respectively. As shown in Figure 11, the pristine sample rapidly decomposes within the 430–530 °C range (DTG peak at approximately 500 °C), with negligible weight loss below 430 °C, indicating excellent thermal stability. After 7 days of immersion in the SC2 solution, the sample exhibited a low-temperature weight loss peak (DTG peak at approximately 170 °C) between 150 and 200 °C. This is attributed to the hydrolysis of the TAIC crosslinking structure under acidic conditions, followed by the release of crystalline water from the hydrolysis products. As the H_2_O_2_ in the solution reacts with the hydrolysis products of TAIC, the low-temperature degradation peak disappears after 28 days of immersion, whereas the main decomposition zone remains largely unchanged. Figure 12 reveals that after immersion in HCl solution, the sample similarly exhibited a low-temperature decomposition peak at 150–200 °C. This peak remained visible after 28 days of immersion, which is consistent with the mechanism underlying the low-temperature degradation peak observed after 7 days of immersion in the SC2 solution. Both phenomena are attributed to the loss of crystalline water from the hydrolysis products of the TAIC cross-linked structure under acidic conditions. As shown in Figure 13, the TG/DTG curves of samples immersed in H_2_O_2_ solution for 7 and 28 days nearly overlap with those of the original sample, indicating no low-temperature weight loss. This finding indicates that no hydrolysis of the TAIC cross-linked structure occurred in the H_2_O_2_ solution. Overall, the primary decomposition zones for samples immersed in all three solutions were concentrated between 430 and 530 °C, with minimal peak temperature variation. The observed low-temperature decomposition is primarily related to chemical changes in the cross-linked structure, which have a negligible influence on the fluorinated main chain. This finding indicates that the material core structure exhibited no significant degradation under either acidic or oxidative conditions, maintaining excellent thermal stability.

**Figure 14:**
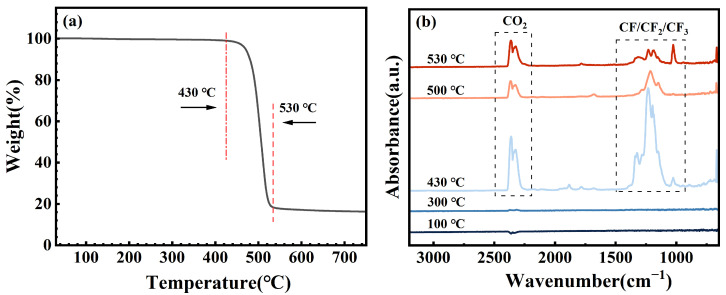
TG-IR analysis of FFKM after aging in the SC2 solution for 28 days, showing (**a**) TG curve and (**b**) IR spectra of gaseous decomposition products.

To further elucidate the structural influence of the SC2 solution on FFKM, TG-IR coupled analysis was conducted on samples aged for 28 days, and the results are presented in Figure 14. As shown in Figure 14a, decomposition commenced at approximately 430 °C, with the decomposition rate peaking at 500 °C. Decomposition is largely complete at 530 °C, indicating high thermal stability. The infrared spectroscopy results (Figure 14b indicate no significant absorption peaks before 430 °C, suggesting that no gaseous products formed. Beyond 430 °C, characteristic absorption peaks for CF, CF_2_, CF_3_, and CO_2_ gradually emerge, reaching a maximum intensity at 500 °C before decreasing with increasing temperature. This pattern aligns with the weight loss phase observed in the TG curve. The CF-type peaks originate from the thermal cleavage of fluorinated main chains at elevated temperatures, whereas the CO_2_ peak arises from the thermal decomposition of C=O and C–O groups within the crosslinked structure. Combining the XPS and FTIR analyses reveals that the synergistic action of HCl acid hydrolysis and H_2_O_2_ oxidation in SC2 partially preserves the C=O and C–O functional groups within the TAIC crosslinking structure. These functional groups undergo further decomposition at elevated temperatures to form CO_2_, confirming that the crosslinked network constitutes the primary degradation region under the influence of SC2, while the fluorinated backbone retains excellent thermal stability.

**Figure 15:**
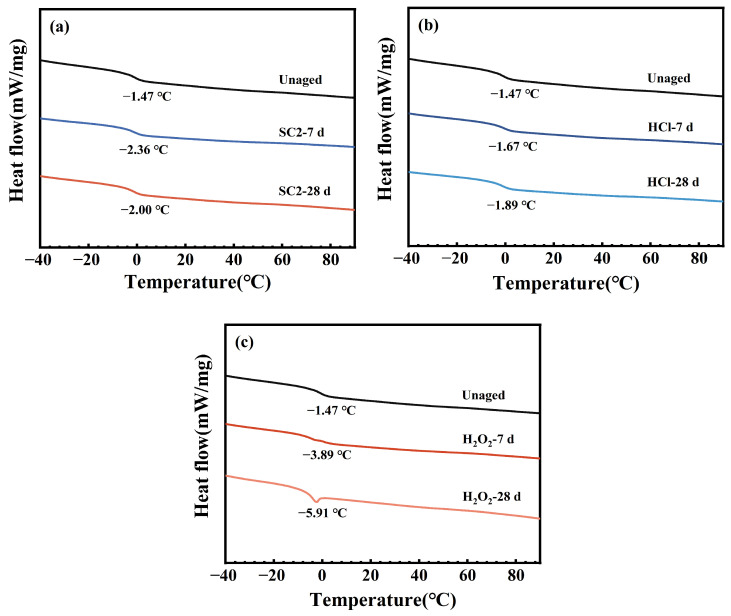
DSC curves of FFKM samples after immersion in (**a**) SC2, (**b**) HCl, and (**c**) H_2_O_2_ solutions.

Figure 15 presents the DSC curves of FFKM after aging in different solutions. The glass transition temperature of a sample can be determined by the abrupt change in heat capacity observed in the DSC curve. The glass transition temperature of the material did not significantly change after immersion in the three solutions, indicating that the main chain structure of the samples remained stable before and after aging, with no apparent degradation occurring.

### 3.5. Cross-Linked Network

**Figure 16:**
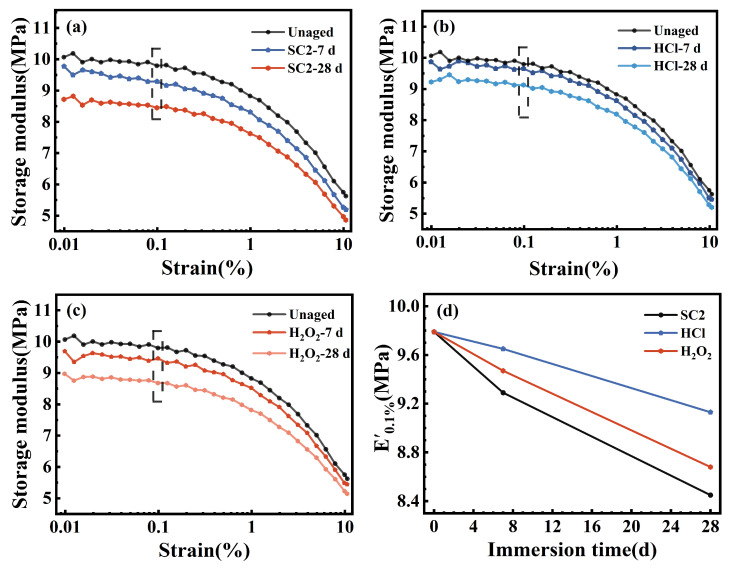
DMA strain scan results of FFKM samples aged in different solutions, including (**a**–**c**) SC2, HCl, and H_2_O_2_ solutions and (**d**) variation in storage modulus at 0.1% strain.

Figure 16a–c display the DMA strain scan results for the FFKM samples after aging in different solutions, whereas Figure 16d illustrates the variation in the storage modulus (E′) at 0.1% strain with immersion time. As evident from Figure 16a–c, E′ remains largely stable (fluctuations < 2%) within the low strain region (<0.2%), indicating that the samples exhibit linear viscoelastic behavior with preserved crosslinking and filler–matrix interactions. Upon exceeding 1% strain, E′ markedly decreases, demonstrating the characteristic Payne effect [34], reflecting polymer chain disentanglement and disruption of filler-filler and filler-matrix networks. As depicted in Figure 16d, E′ at 0.1% strain consistently decreased with prolonged aging duration. In the SC2, HCl, and H_2_O_2_ solutions, E′ decreased from 9.79 MPa to 8.45, 9.13, and 8.68 MPa, respectively, with the order of reduction being SC2 > H_2_O_2_ > HCl. This finding indicates that the synergistic action of acidity and oxidation caused the most pronounced degradation of the crosslinked network in SC2. As E′ is positively correlated with crosslink density at low strains, this trend indicates that all three solutions induce a time-dependent reduction in crosslink density. The most pronounced weakening of the crosslinked structure occurred in the SC2 environment, further corroborating the accelerated aging and synergistic degradation effects of HCl and H_2_O_2_ within the SC2 solution.

### 3.6. Mechanical Properties

**Figure 17:**
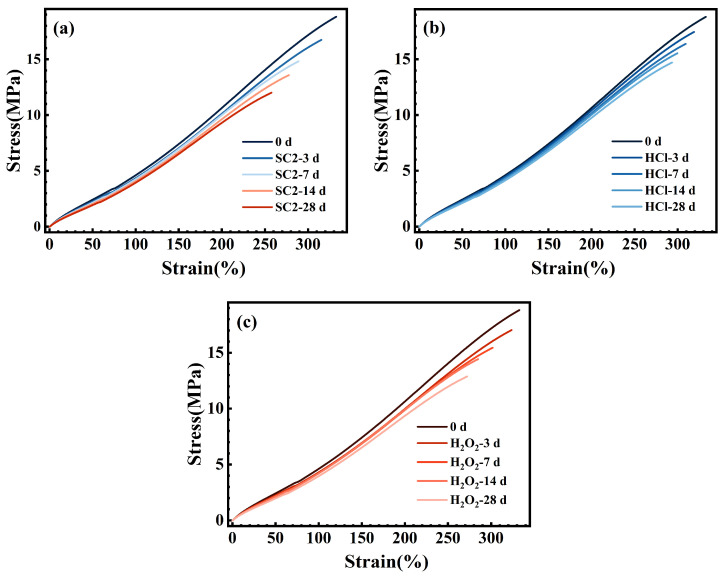
Stress–strain curves of FFKM samples after immersion in (**a**) SC2, (**b**) HCl, and (**c**) H_2_O_2_ solutions.

Sealing components in practical applications are not solely subjected to compression; they also endure assembly stretching, localized stretching caused by vacuum or pressure differentials, and the associated risk of tearing [35,36]. Concurrently, chemical aging induces significant alterations in the tensile properties of sealing materials. This may precipitate cracking during assembly, opening/closing operations, or localized edge gripping, ultimately manifesting as ‘seal failure’ [22,37]. Consequently, evaluating structural integrity and damage resistance post-chemical aging through tensile strength holds significant engineering relevance for sealing applications. Moreover, tensile properties serve as a sensitive indicator for assessing the integrity of crosslinked networks after chemical/thermal aging, linking changes in microstructure to macroscopic performance [38,39]. Consequently, tensile tests were conducted on FFKM before and after immersion in three solutions to reflect tensile property variations, with results shown in Figure 17. The figure demonstrates that FFKM exhibits typical non-linear tensile response characteristics of rubbers above their glass transition temperature in all three solutions before and after aging. The overall slope of each curve decreases with increasing aging time, indicating that the material evolves from a higher initial modulus toward a lower stiffness, i.e., from a ‘hard and brittle’ state to a ‘soft and tough’ state. This trend is most pronounced in SC2, followed by H_2_O_2_, with the weakest effect observed in HCl. This reflects differences in the extent to which various media weaken the cross-linked network and the filler-matrix interface.

**Figure 18:**
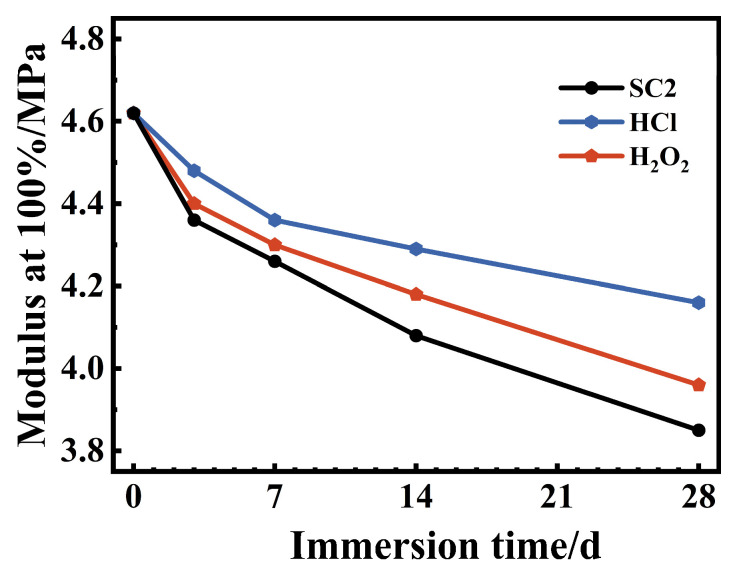
Variation in modulus at 100% of FFKM after immersion in different solutions.

Figure 18 shows the variation curves of the 100% elongation strength of FFKM after aging in three solutions for different durations. The 100% elongation strength (M100) serves as an indicator of cross-linked network density under low-strain conditions. The initial sample exhibited an M100 value of 4.62 MPa, which decreased to 4.26 MPa (after 7 days) and 3.85 MPa (after 28 days) in SC2; to 4.36 MPa (after 7 days) and 4.16 MPa (after 28 days) in HCl; and to 4.47 MPa (after 7 days) and 3.96 MPa (after 28 days) in H_2_O_2_. The order of reduction was SC2 > H_2_O_2_ > HCl, which was consistent with the DMA results. This further demonstrates that the concurrent action of acidity and oxidation significantly disrupts the cross-linked network in the SC2 environment, followed by H_2_O_2_, whereas the impact of a single acidic environment is comparatively minor.

**Figure 19:**
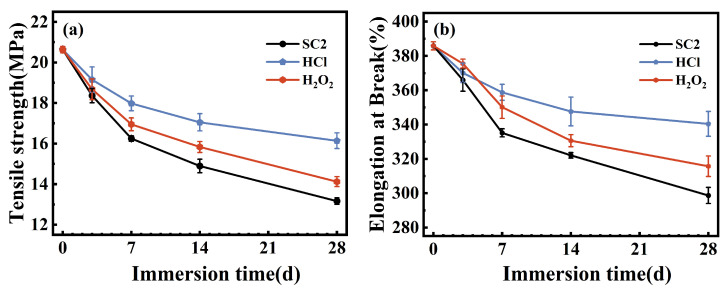
Variations in the tensile properties of FFKM after immersion in different solutions: (**a**) tensile strength and (**b**) elongation at break.

Figure 19 shows the changes in tensile strength and elongation at break for the FFKM samples after immersion in the three solutions. As shown in Figure 19a, the tensile strength of the material monotonically decreased with aging time in all three solutions: from 20.6 MPa (0 days) to 13.1 MPa (SC2, 28 days), 14.2 MPa (H_2_O_2_, 28 days), and 16.1 MPa (HCl, 28 days), with a similar decrease following the order SC2 > H_2_O_2_ > HCl. The elongation at break also decreased in a similar sequence: from 385.88% (0 days) to 298.69% (SC2, 28 days), 315.72% (H_2_O_2_, 28 days), and 340.45% (HCl, 28 days). The concurrent decrease in strength and ductility indicates that aging primarily occurs at the cross-linking points and their surrounding TAIC-related structures, leading to a reduction in the effective network density and interfacial constraint.

### 3.7. Degradation Mechanisms of FFKM in Acidic SC2 Solution

**Figure 20:**
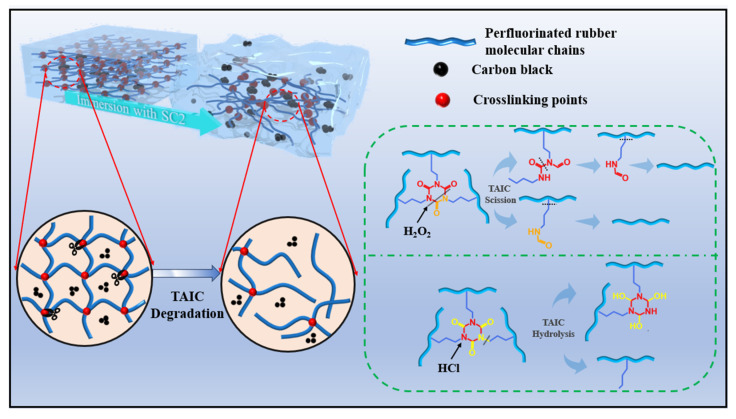
Degradation mechanism of FFKM in acidic SC2 solution.

In summary, the performance changes observed in FFKM following exposure to the SC2 solution are attributable primarily to alterations in its cross-linked structure, whereas the main chain remains relatively stable with no significant changes. As illustrated in Figure 20, the alterations observed in FFKM within the SC2 solution result from the combined action of HCl and H_2_O_2_ solutions. Both solutions contribute to the degradation of the cross-linked structure, thereby inducing changes in the properties of FFKM. Under the influence of the HCl solution, the TAIC cross-linked structure undergoes hydrolysis in a high-temperature acidic environment, where the oxygen atom on the ring becomes protonated, causing the N–C(=O)–N bond to react and form an N–C(–OH)–N bond, thereby disrupting the cross-linked structure. Additionally, the C–N bonds connected to the TAIC ring structure may also break, leading to destruction of the cross-linked network. In the HCl solution, both types of disruption may occur simultaneously. In H_2_O_2_ solution, the C–N bonds within the ring structure of the TAIC cross-link react with the H_2_O_2_ solution, causing C–N bond cleavage and ring opening. With prolonged immersion, all the C–N bonds on the TAIC undergo cleavage, destroying the cross-linked structure. Concurrently, the H_2_O_2_ solution also cleaves C–N bonds outside the TAIC ring structure. Furthermore, alkyl chains attached to the FFKM backbone may also fracture, leading to destruction of the cross-linked network while the backbone itself remains stable. In the SC2 solution, these two solutions exhibit synergistic effects: the hydrolytic action of HCl enhances solution penetration and exposes reaction sites, accelerating H_2_O_2_ oxidation of TAIC. This hastens the depletion of cross-linking sites and network relaxation, increasing the reaction rate of acid hydrolysis and causing severe disruption of the FFKM cross-linked structure in the SC2 solution. Concurrently, during immersion, the weakened bond between the filler and the matrix due to cross-linked disruption causes filler release from the sample, altering the FFKM properties. During aging, FFKM samples exhibit diminished mechanical properties that progressively deteriorate with immersion duration. This degradation stems from both cross-link breakage and filler release.

## 4. Conclusions

The degradation behavior and mechanisms of a TAIC-cross-linked FFKM in SC2, HCl, and H_2_O_2_ solutions were systematically investigated. The results indicate that all three solutions cause significant sample weight gain and performance degradation, with the most pronounced effects observed in the SC2 solution. This is primarily attributed to the synergistic action of acid hydrolysis (HCl) and oxidative chain scission (H_2_O_2_) within the same system. Under HCl solution action, hydrolysis occurs at the C–N bond connected to the TAIC ring and the N–C(=O)–N bond within the TAIC ring structure. This process enhances solution permeation and exposes reactive sites, thereby intensifying the subsequent destruction of C–N bonds in the TAIC structure by the H_2_O_2_ solution; simultaneously, the oxidative action of the H_2_O_2_ solution accelerates cross-link depletion and network relaxation, thereby increasing the reaction rate of acid hydrolysis. In summary, the degradation of FFKM in SC2 solution primarily stems from the destruction of the cross-linked network under the synergistic action of HCl and H_2_O_2_, while the polymer backbone and side groups remain largely intact. As the cross-linked network deteriorates, the matrix’s binding force on fillers significantly weakens, leading to filler detachment and void formation on the material surface, ultimately leading to a substantial decline in mechanical properties. It should be emphasized that the 28-day test conducted in an 85 °C SC2 solution may be regarded as a long-term simulation under actual RCA operating conditions. This test amplifies potential failure modes arising from cumulative multiple wash cycles, thereby establishing a direct correlation between experimental outcomes and real-world RCA usage scenarios. It is evident that the critical vulnerability of FFKM in acidic peroxide SC2 lies in the sensitivity of the TAIC crosslink bonds to acid/oxidation and the insufficient interfacial confinement force resulting from reduced crosslink density.

## Data Availability

The original contributions presented in this study are included in the article. Further inquiries can be directed to the corresponding author.
